# Severe dementia: A review about diagnoses, therapeutic management and ethical issues

**DOI:** 10.1590/S1980-57642010DN40300003

**Published:** 2010

**Authors:** Lilian Schafirovits-Morillo, Cláudia Kimie Suemoto

**Affiliations:** 1 Disciplina de Geriatria, Departamento de Clínica Médica do Hospital das Clínicas da Faculdade de Medicina da Universidade de São Paulo, São Paulo SP, Brazil.

**Keywords:** dementia, Alzheimer’s disease, severe

## Abstract

North American data show that in the year 2000 around 4.5 million people had a diagnosis of dementia and more than a half were at moderate or severe stages of the disease. There is inevitable cognitive and functional decline caused by all etiologies of irreversible dementia as well as many behavioral symptoms that compromise the quality of life of both patients and caregivers. Few published studies have investigated issues concerning severe dementia such as predictors of mortality and life expectancy, nutrition, end of life issues and palliative care in terminal dementia, as well as best pharmacological and non-pharmacological treatments. Due to the complexity that characterizes advanced dementia, it is important that this discussion starts as early as possible allowing some decisions to be taken, preferably when the patients can still express their opinion.

## Abstract (pt)

Dados Norte-Americanos apontam que o contingente de portadores de DA no ano 2000 era de cerca de 4,5 milhões de pessoas e são esperados em torno de 13 milhões para o ano 2050 sendo mais da metade em fase moderada e grave da doença. Além do declínio cognitivo e funcional, são muito comuns diferentes sintomas comportamentais, que muito diminuem a qualidade de vida de pacientes e cuidadores. São escassos os estudos para o desenvolvimento de fármacos, conhecimento de medidas não-farmacológicas, estudos epidemiológicos que averiguam fatores que interferem na história natural da doença, formas de abordar o fim de vida, além de questões como nutrição e linguagem. O longo curso desta doença de prognóstico temporal incerto traz consideráveis dúvidas quanto às decisões a serem tomadas. A visão do conforto e proporcionalidade das ações e do princípio do não-malefício podem servir de guia para a tomada de decisão.

North American data show that in the year 2000 around 4.5 million people had a diagnosis of dementia and more than a half were at moderate or severe stages of the disease (31% moderate and 21% severe phase).^1^

Mean survival after dementia diagnosis varies between one to 16 years, whereas one third of demented individual live to advanced stages.^2^

Because of the inevitable cognitive and functional decline caused by all etiologies of irreversible dementia, geriatricians, neurologists and psychiatrists face the challenge of providing patients and their caregivers with the best care as well as to communicate responsibly and truthfully their real expectations about the progressive course of the disease.

During the course of progression of dementia, patients gradually lose independence and autonomy while the severe phase is characterized by loss of capacity to provide self care in basic activities of daily living, such as eating, bathing and walking independently. In this stage there are also many behavioral symptoms that compromise the quality of life of both patients and caregivers and are sources of great stress and burden to the latter with institutionalization being the ultimate consequence.

Despite these relevant data, few trials have investigated issues concerning severe dementia in the medical literature such as predictors of mortality and life expectancy, nutrition, end of life issues and palliative care in terminal dementia, among others. The difficulty in measuring responses to interventions can explain to some extent the lack of interest in these phases but since the 1990s instruments have been developed in this regard as we will discuss further in this paper.

## Disease progression and life expectancy – predictors

A variety of factors interfere in the progression of dementia, modifying its course. The rhythm of cognitive and functional decline as well as the length of time of the disease and the survival of the patients are not uniform and instead vary depending on the etiology of dementia, the comorbidities of the patient and the quality of care provided by the health team and caregivers.

Larson et al.^3^ conducted a prospective study between 1987 and 1996 to determine some of these factors and found that age above 85, gait disorders, wandering and comorbidities such as diabetes and heart failure were related to significantly lower survival of patients.

Other factors associated to shorter survival were male gender, lower score on the Mini-Mental State Examination (MMSE) at the initial evaluation, greater functional disability, presence of extrapyramidal signs and history of falls, arterial coronary disease, stroke and urinary incontinence. Rapid cognitive decline, defined by the loss of 5 or more points on the MMSE during one year of follow up, was also associated with lower survival.

There are insufficient data regarding prognostic factors in advanced dementia, such as the presence of neuropsychiatric symptoms, quality of care, nutritional status and caregiver burden. This knowledge would facilitate the planning of prevention and treatment of modifiable conditions and the establishment of a palliative approach when necessary.

## Cognitive and functional evaluation in severe dementia

The assessment instruments allow the disease to be classified into stages, and enables monitoring of the progression of symptoms and the response to therapeutic intervention. The ideal evaluation of moderate and severe dementia assesses cognitive, functional and behavioral symptoms as well as caregiver burden.

The most widely used cognitive assessment instrument, both in practice and in clinical trials is the MMSE.^4^ Scores between 11 and 17 suggest moderate stage of disease while scores less than or equal to 10 indicate advanced stage. This test has a floor effect for severely demented subjects and so new tools were developed to better monitor these patients.

In 1990, the Severe Impairment Battery (SIB) was developed.^5^ The scale’s score ranges from zero to one hundred points and provides an accurate approach to changes in cognition over time in patients with MMSE scores below 15 points. It addresses challenges of low complexity, reflecting the severity of the dementia. The scale scores partially correct responses as well as non-verbal interaction and uses simple language to facilitate understanding by patients. Ratings below 63 indicate well advanced dementia cases. The instrument was able to demonstrate the benefit of the receptor antagonist N-methyl-D-aspartate (Memantine) on cognition in patients with moderate and severe Alzheimer’s disease.^6^

Besides cognitive evaluation, dementia stratification can be assessed by scales that include functional performance of patients, such as the Global Deterioration Scale (GDS) and Functional Assessment Staging (FAST).^7,8^ Another scale, called the Clinical Dementia Rating (CDR) scale, allows for a cognitive and functional approach.^9^ Table 1 summarizes the main features of these assessment tools.

**Table 1:** Assessment tools in dementia.

Scale	Domain	Score	Interviewee	Comments
MMSE	Cognition	0 to 30	Patient	Floor effect in severe dementia
CDR	Global status	0 to 3	Patient and caregiver	Scale extended to 4 (profound dementia) and 5 (terminal dementia)
GDS	Global status	0 to 7	Patient and caregiver	Limited use in severely demented patients
FAST	Functional status	0 to 6 (A to E) and 7 (A to G)	Caregiver	Very useful in advanced dementia
SIB	Cognition	0 to 100 (<63: severe impairment)	Patient	Very useful in advanced dementia
Severe MMSE	Cognition	0 to 30	Patient	Shorter than SIB
ADCS-ADL	Functional status	0 to 54	Caregiver	Modified from the original scale applied to less severe patients

MMSE: Mini Mental State Exam; CDR: Clinical Dementia Rating; GDS: Global Deterioration Scale; FAST: Functional Assessment Staging; SIB: Severe Impairment Battery; ADCS-ADL: Alzheimer's Disease Cooperative Study-Activities of Daily Living.

The incorporation of these instruments into clinical trials allowed the rate of worsening or improvement of patients to be quantified in advanced stages. Moreover, they permitted better stratification of this long phase known as severe dementia, which included patients with very different cognitive, behavioral and functional profiles. Thus, among patients classified as CDR 3, known as severe dementia, there are individuals who still communicate verbally and walk without support, but also others who are confined to bed, unable to sustain their head or already in the fetal position. The FAST scale in our experience is the most accurate instrument for classifying patients with advanced dementia.

The advantages of better classification of advanced dementia include the possibility of better studies designed to validate interventions in cognition, behavioral and functional status, as well as better approaches in terminal dementia using palliative care. Therefore, we can assume that the evaluation tools for advanced dementia have at least two main goals:

1. To classify patients into more homogeneous groups in order to better understand the validity of the interventions in each specific segment;
2. To monitor the response of improvement or deterioration in cognition, behavior and functionality of these individuals.

## Management of patients in advanced stages of dementia

Patients with advanced dementia should be monitored closely by health care teams. As their disease progresses, these patients become increasingly frail, their condition may change rapidly and they may exhibit behavioral and psychological symptoms more frequently.

The Third Consensus Conference on Diagnosis and Treatment of Dementia devoted a section to the advanced stage of disease. It was recommended that patients should visit their doctors every three months when they are medicated and every four months when not.^2^ The Consensus also recommends that patients are evaluated for cognition, behavior, functional, nutritional and clinical status. Furthermore, guidelines on the prevention of falls and caregiver burden are emphasized.

Caregivers are an integral part of the healthcare team and the expectations of treatment should be realistically presented and discussed with them. In addition, topics concerning end of life must be dealt with according to plans made by the patients or according to the will of their caregiver, in line with current legislation and best professional awareness of the health team.

In the Advanced Cognitive Impairment Outpatient Clinic (Department of Geriatrics, University of São Paulo, School of Medicine), patients are evaluated every 1 to 4 months, depending on their medical and behavioral condition. The instruments used for cognitive assessment are the MMSE and the Severe MMSE.^10^ The SIB scale is used for patients in advanced stages. The test of memory of pictures, semantic and phonemic fluency and the Clock Drawing Test are used in patients with moderate dementia. The Clinical Dementia Rating Scale (CDR) is utilized to classify the severity of dementia. For the functional classification, the FAST scale is applied in all patients and the Pfeffer^11^ scale only for those patients at the moderate stage of the disease. In the behavioral evaluation, we use the Neuropsychiatric Inventory^12^ and the Cornell scale for depression in dementia.^13^ In addition, patients are evaluated by a team of speech therapists regarding communication and swallowing issues.

There are many challenges in relation to providing the best care to advanced dementia patients and their families, and many questions remain unanswered by the medical literature. Efforts should focus on using the best evidence currently available and developing well-designed studies to clarify with clear scientific evidence the doubts that still remain.

## Principles of treatment

Even in advanced stages of dementia, there are interventions that can lessen the impact of the disease for patients and their caregivers. Some fundamental principles can be useful in the management of patients with advanced dementia:^14^

1. **Something can be done:** It is known that relatively simple interventions can have a big impact on symptoms and patient functionality. For example, decreasing the dose of a medication or changing the schedule of administration may improve cognitive or behavioral symptoms. Identifying these opportunities is part of advanced demented patient care.
2. **The diagnosis matters:** The initial evaluation should always be comprehensive, even when performed in advanced stages of disease. How and when do the symptoms begin? What is the pattern of behavioral, cognitive and functional changes? What was the previous response to therapy? What is the specific diagnosis of dementia? The answers to these questions influence patient management.
3. **The disability presented by the patient is multifactorial:** Clinical comorbidity, sensory deficits, side effects of medications, environmental stressors, caregiver variables and the etiology of cognitive decline work together in the cognitive, functional and behavioral deficits in dementia.
4. **Patients with dementia have residual abilities:** It is common to ignore that the functions which are still preserved are as important as those that were lost. Is the ability to walk, feed themselves independently or to respond to social stimuli still preserved? A care plan should be implemented so that the autonomy and quality of life of patients can be optimized.
5. **Emotions and needs of the patients should not be overlooked:** Even in advanced stages of dementia, the ability to communicate and understand emotions is often maintained, a phenomenon known as affective preservation. Enhancing the emotional state of patients and their relatives may facilitate adherence to treatment.
6. **The patient and family are a unit:** The family is a valuable source of information about health history, personality and characteristics of the patient. Moreover, it has a key role in monitoring and implementing the intervention planned.

## Management of behavioral and psychological symptoms

Psychiatric and behavioral symptoms are characteristic of moderate and advanced dementia.^14^ When they are present they lead to a great caregiver burden, principally when agitation and aggressivity are present and may often precipitate patient institutionalization.^15^ Interventions are important because these symptoms respond more quickly than cognitive or functional deficits.

The first step in the evaluation of behavioral symptoms is the exclusion of environmental stressors and clinical events that can cause or at least can contribute to the observed symptoms. It must be presumed that behavioral changes are secondary to delirium until proven otherwise. Side effects of newly introduced drugs should be evaluated. Complete physical examinations and drugs reevaluation should be carefully carried out. The laboratorial investigation to exclude causes of delirium includes complete blood count, electrolytes, calcium, glucose and urinalysis.^2^

Social factors can be the trigger of behavioral symptoms. The interaction with the caregiver can provoke resistance and agitation, depending on how the patient is addressed. Moreover, pain assessment should be done carefully because pain is difficult to recognize in patients with advanced speech disorders.

The short term use of physical restraint may be necessary until the adopted interventions show efficacy, principally when there is considerable risk for the patients or their relatives. However, the chronic use of physical restraint is highly discouraged because it can cause muscular atrophy and contraction, pressure sores, thrombosis and worsening of agitation.

## Non-pharmacological treatment

Non-pharmacological approaches should be considered before pharmacological interventions. Although non-pharmacological treatments have not been systematically tested in advanced dementia, some therapies have been tested in other stages of AD through the use of randomized clinical trials in some studies.^16^

Behavioral treatment for depression, music therapy, controlled multi-sensory stimulation and pet therapy are some of the methods used to control behavioral and psychological symptoms in dementia, such as agitation, apathy and depression.^16^ However, the effect of these treatments seems to be limited to the period of sessions, with no significant benefits when discontinued.^17^

On the other hand, educational programs and support for caregivers seem to be effective over the long term. In these programs, caregivers receive information about the disease and instructions on how to deal with behavioral symptoms. Caregivers can also share previous experiences that can influence other individuals who are experiencing a similar situation. In Brazil, the Brazilian Association of Alzheimer (ABRAZ) organizes briefings and gives support for caregivers of patients with dementia.

Despite the lack of consistent scientific evidence, discrete benefits and the frequent lack of sustainable effects, non-pharmacological interventions should be the first choice to treat behavioral symptoms due to their higher safety profile compared to pharmacological treatment.^2^

## Pharmacological treatment

### Behavioral and psychological symptoms in dementia

Behavioral and psychological symptoms such as agitation, aggression, psychosis, apathy and depression tend to respond to pharmacological treatment, while wandering and inappropriate sexual behavior often do not respond. Symptoms such as severe depression, psychosis and aggression should be treated with medication as the first line, along with non-pharmacological measures, in view of the risk that these symptoms pose to the patients and their caregivers.^2^

Some randomized clinical trials using atypical antipsychotics to treat behavioral and psychological symptoms in severe demented patients have been performed.^18^ Evidence suggests that risperidone, olanzapine and quetiapine are more effective than placebo. However, in recent years, the use of atypical antipsychotics has been associated with increased risk of cerebrovascular events and increased mortality in randomized trials.^18^ A meta-analysis of 15 randomized trials concluded that there is a significantly increased risk of death (OR=1.54, 95% CI 1.06 to 2.23, p=0.02) when atypical antipsychotics are used.^19^ However, alternatives to the use of atypical antipsychotics include typical antipsychotics, such as haloperidol, which have a similar or perhaps even worse risk profile than the atypical drugs.^20^ Thus, the need for antipsychotics should be carefully assessed and these drugs should be used only in cases of severe agitation, aggression and psychosis involving risk to patients and their caregivers. After three months of stability of behavioral symptoms, it is advisable to reduce and discontinue the antipsychotic. Evidence shows that this procedure can be done without significant exacerbation of symptoms.^2^

Other medications may also be used to treat agitation and aggression. The antidepressants citalopram and trazodone and anticonvulsants carbamazepine and valproic acid are used to control these symptoms, but less effectively than antipsychotics.^21^ Benzodiazepines have also been studied in clinical trials. The best evidence of efficacy and safety was with the short-term use of lorazepam for acute agitation. However, due to side effects such as falls, excessive sedation, worsening of cognition, tolerance and dependence, these medications should only be used in emergencies or as a sedative during procedures.^2^

Another challenging problem in advanced dementia is to assess the presence of depression. The diagnosis of depression in patients who have limited verbal communication skills is complex. The presence of depressive symptoms, isolation and/or irritability may be treated with antidepressants, even in patients with advanced dementia. The use of serotonin reuptake inhibitors is recommended because of their efficacy and safety in patients with mild and moderate AD.

**Figure 1:**
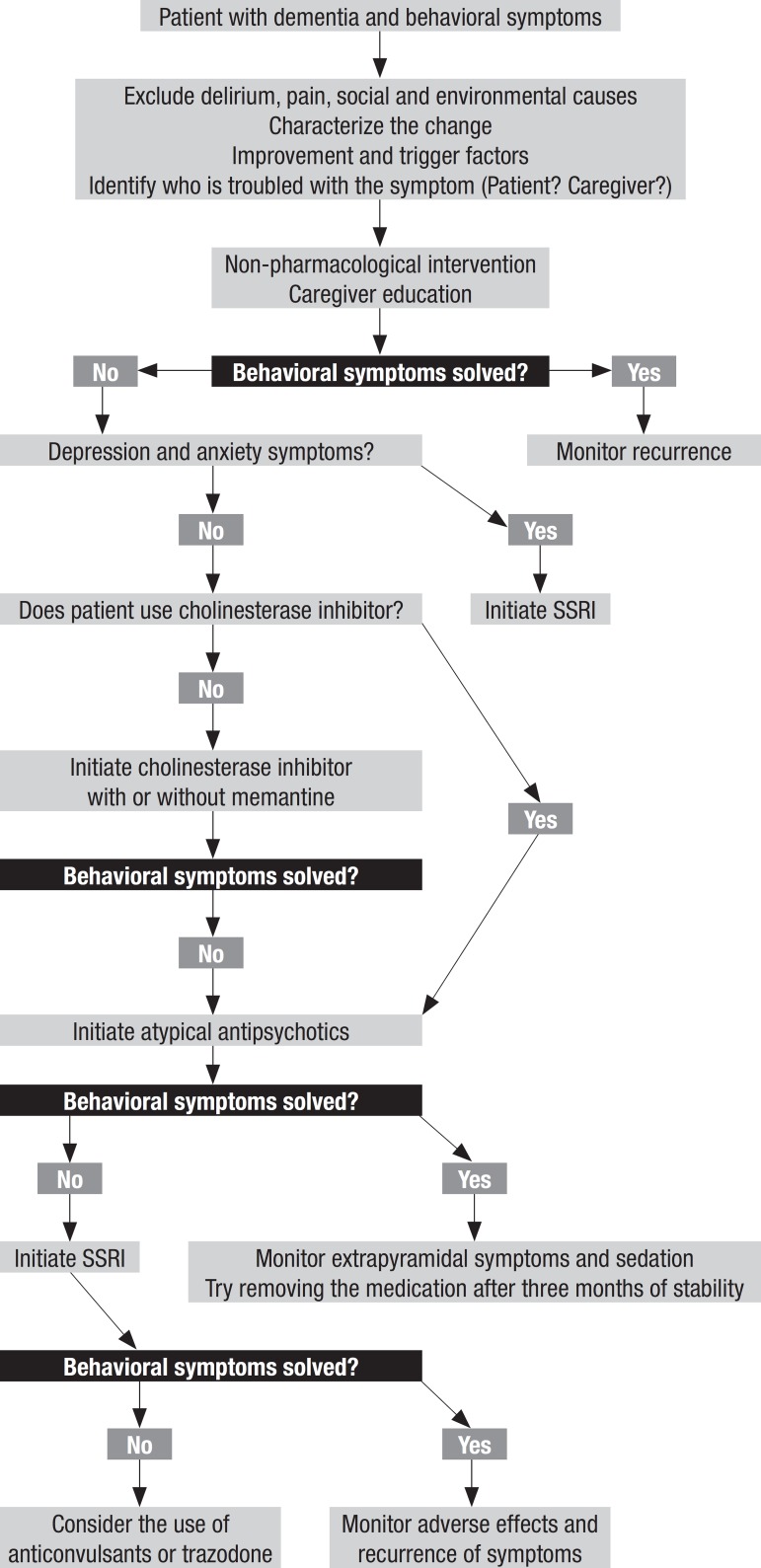
Algorithm for management of behavioral and psychological symptoms in patients with advanced dementia (Adapted from Sink et al.).^21^

An algorithm for the management of patients with behavioral and psychological symptoms in dementia is suggested in Figure 1.

### Cognitive decline

Pharmacological interventions to improve cognition in patients with advanced dementia include cholinesterase inhibitors and memantine. Three randomized trials using cholinesterase inhibitors in patients with moderate to advanced dementia^22-24^ and two other studies involving only patients with severe dementia^25,26^ showed that this class of medication improves cognition, function and behavior even in advanced stages of disease. A recent Cochrane review on the use of anticholinesterasics in AD suggests that the effects observed in patients with advanced dementia are similar to those observed in mild and moderate demented subjects.^27^ Therefore, the anticholinesterase drugs should be recommended even in advanced stages of dementia, although the benefits are modest.^2^

Despite the observed benefits in cognition and function, there is no evidence that the use of cholinesterase inhibitors can delay institutionalization of patients with advanced dementia. Relative contraindications to the use of this medication include cardiac conduction defects (except for right bundle branch block), severe chronic obstructive pulmonary disease and previous history of peptic ulcer disease without the use of cytoprotective agents. The most common adverse effects are gastrointestinal and include anorexia, nausea, vomiting and diarrhea.^2^

Memantine is also an option for improving cognition in patients with advanced dementia. Four randomized trials with memantine involved patients with moderate and severe dementia.^28-31^ Tariot et al.^29^ compared the use of memantine and placebo in patients with severe dementia who were already in use of donepezil, demonstrating an additional improvement in cognition with the use of memantine. There is evidence that memantine improves cognition, function and behavioral symptoms such as agitation and aggression. It was shown that it would be necessary to treat six patients with memantine for improvement or stabilization of general measures in one patient.^31^

There is scant data showing the most appropriate time to stop treatment with anticholinesterase or memantine. The cited studies included patients with scores on the MMSE from 3 to 5, although patients with scores lower than these may also benefit from treatment. Thus, treatment should be continued until the clinical benefits cannot be further evaluated. Patients who are bedridden or in mutism represent a major challenge to cognitive and functional assessment and should be candidates to stop therapy.

Besides the improvement or stability of the symptoms, even slower rates of decline can be seen as a positive aspect of treatment. Thus, before and after the introduction of a new intervention, whether pharmacological or not, the cognition and the functional status of the patient should be tested. If the rate of decline is faster than expected, the medication should be discontinued. In this case, withdrawal symptoms and worsening of cognition may occur. Thus, the patient should be carefully monitored after the withdrawal of these medications.^2^

### Therapeutic decisions

The long course of this disease, which has an uncertain temporal prognosis, raises considerable doubts regarding the decisions to be taken. Introduction or otherwise of alternative ways of feeding, antibiotics for repeated infections, measures of advanced life support, hospitalization in intensive care units are the most frequent questions which the families and the team have to deal with. Also, long term institutionalization also generates a lot of questions and mixed feelings, involving relief and guilt in family members.

Ethical and religious values of patients and their families make up the network of variables that must be considered when each issue is tackled. The idea of comfort, proportionality of actions and the principle of non-harm (“primum in nocere”) that guides the practice and ethics of Palliative Medicine can serve as a guide for decision making. In the quest for dignity and comfort of the patients and their families, curative and palliative measures should be balanced.^14^

Due to the complexity that characterizes advanced dementia and its various medical and ethical dilemmas, it is important that this discussion starts as early as possible and allowing some decisions to be taken, preferably when the patients can still express their opinion.^32^ Thus, it is possible for decisions to be taken more rationally, so that decisions are the result of reflection and not of ambiguous feelings common in extreme situations when family members are called upon to opine on matters for which they feel unprepared.

Cultural differences on this issue are relevant. However, national studies are very scarce in the literature. It is important to know what the Brazilian population beliefs are regarding the initial approach to issues of advanced dementia so that strategies can be formulated to reflect the interests of patients and their relatives.
